# Public Health Word of the Year 2022 – New Normal

**DOI:** 10.1002/puh2.102

**Published:** 2023-07-03

**Authors:** Don Eliseo Lucero‐Prisno, Deborah Oluwaseun Shomuyiwa, Sheena Ramazanu, Yusuff Adebayo Adebisi, Jude Altiveros Duarte, Patrick Alain Azanza, Martin C. S. Wong, M. B. N. Kouwenhoven

**Affiliations:** ^1^ Department of Global Health and Development London School of Hygiene and Tropical Medicine London UK; ^2^ Faculty of Management and Development Studies University of the Philippines Open University Los Baños Laguna Philippines; ^3^ Faculty of Public Health Mahidol University Bangkok Thailand; ^4^ Faculty of Pharmacy University of Lagos Lagos Nigeria; ^5^ Leadership Institute for Global Health Transformation, Saw Swee Hock School of Public Health National University of Singapore Singapore Singapore; ^6^ Nuffield Department of Population Health University of Oxford Oxford UK; ^7^ Southern Leyte State University Sogod, Southern Leyte Philippines; ^8^ Catanduanes State University Virac Catanduanes Philippines; ^9^ JC School of Public Health and Primary Care, Faculty of Medicine Chinese University of Hong Kong Hong Kong SAR China; ^10^ Department of Physics Xi'an Jiaotong‐Liverpool University (XJTLU) Suzhou China

**Keywords:** COVID‐19, global health, health systems, new normal, pandemic, public health

## Abstract

The COVID‐19 pandemic caused significant disruption and forced the global community to adapt to a new way of life. The term ‘new normal’ encapsulates the notion that the pandemic has triggered radical changes, requiring rapid adaptation from the global community. The article focuses on examining the practical implications of the new reality in 2022, the challenges it presented, and the opportunities it offers for shaping the future of global health. The article also highlights significant public health stories that emerged in 2022, including the mpox surge, the impact of climate change, and reproductive health issues and their impact in shaping the ‘new normal’. It argues that the ‘new normal’ provides a unique opportunity to rebuild our world more responsively and sustainably. It emphasizes the need for global communities to maintain adaptability and foster innovation in the face of ongoing challenges. Making informed choices becomes crucial for achieving success within this shifting landscape. Conclusively, the article advocates for embracing the ‘new normal’ as a catalyst for positive change, both individually and collectively. By doing so, global communities can navigate the challenges ahead and create a world that is better prepared for future health crises.

## INTRODUCTION

The field of public health is constantly evolving, as new challenges and emerging issues require innovative solutions. The COVID‐19 pandemic has caused significant disruption over the last 3 years. As the world retires lockdowns and restrictions, new processes, programs and systems have been incorporated to deal with existing and new challenges. In 2022, COVID‐19 continued to make headlines with news about boosters, new variants and research on long COVID [1]. Other news, such as mpox's spread, climate change's impact and reproductive health, emerged in the year [2]. It is increasingly clear that the current global health landscape differs from the former years. The global community has experienced a fundamental shift and restructuring across all sectors. The year 2022 saw the emergence of the term ‘new normal’ as a ubiquitous expression during the COVID‐19 pandemic and the subsequent public health response [3]. The concept ‘new normal’ has fluidly adapted to discoveries of novel systems and assumptions that were previously taken for granted. The concept represents a critical idea with profound implications for public health and society.

As the global community emerges from the pandemic, we have a unique opportunity to rebuild our world more responsively and sustainably. From mask‐wearing and social distancing to remote work and telemedicine, the pandemic has fundamentally altered the way we live and interact with each other. As we reflect on the global health challenge, it is no surprise that the ‘new normal’ has been elected as the public health word of the year 2022. This commentary will explore the ‘new normal’ concept and its significance for public health and society in 2022. We will examine the practical changes that have emerged from this representation, the challenges and the opportunities for the ‘new normal’ to shape the future of global health.

## THE ‘NEW NORMAL’ IN CONTEXT

The concept of the ‘new normal’ emerged during the Global Financial Crisis (GFC) in 2008, which brought about economic turmoil and social unrest, fundamentally altering various aspects of human life [3]. The crisis generated uncertainty and disrupted familiar ways of living, prompting a longing for a return to pre‐crisis or ‘normal’ times. During the GFC, individuals re‐evaluated their consumption patterns, opting to save more, spend less and adopt simpler lifestyles in response to the shrinking economy [4]. In times of crisis, there is often an opportunity to collectively shape a better society and adapt to the ‘new normal’. Adapting resiliently to challenges and embracing the ‘new normal’ becomes crucial in effectively addressing complex societal issues.

In 2022, a year filled with numerous events, the concept of the ‘new normal’ evolved to adapt to the ever‐changing complexities of the COVID‐19 pandemic. Future historians may regard 2022 as a pivotal moment in history, marking the end of one era and the beginning of another. Despite hopes for the eradication of the virus, COVID‐19 persisted as one of the most prevalent respiratory viruses in society [5]. Although the Omicron variants and their sub‐variants drove the number of cases higher at the start of the year, significant milestones were achieved in COVID‐19 prevention and treatment [6, 7]. The availability of vaccines for younger populations, routine at‐home testing, and the introduction of bivalent vaccines to address new strains contributed to the reduction of COVID‐19 morbidity throughout the year.

As the impact of the COVID‐19 pandemic eased in several countries, stringent measures implemented to manage the pandemic were gradually retired and replaced with new public health categorizations and blended systems, allowing for the cautious resumption of activities. These categorizations employed colour‐coded or tiered systems to classify regions based on COVID‐19 risk levels, taking into account infection rates, hospital admissions and community indicators [8]. From strict lockdowns in high‐risk areas to relaxed guidelines in low‐risk areas, the categorizations aimed to strike a balance. Blended systems, incorporating both in‐person and virtual activities, became prevalent in sectors, such as education and workplaces.

Throughout 2022, significant public health stories unfolded, shedding light on valuable lessons learned from the COVID‐19 pandemic regarding the importance of urgency in of health system responses. The outbreak of mpox and the resulting rise in stigma against queer men prompted swift and decisive measures to address information management, access to testing and treatment [7]. Additionally, notable public health concerns in 2022 included the amplified impact of climate change, oral health issues, the escalating prevalence of vaping and tobacco use among young individuals and the reproductive health predicament in post‐Roe America [2]. These issues underscored the importance of addressing broader public health issues beyond the immediate pandemic response. The year was further marked by a permacrisis and characterized by various conflicts and humanitarian crises, including the food crisis, Iranian protests and the Russian invasion of Ukraine [7]. These events highlighted the interconnectedness between public health and geopolitical challenges, underscoring the need for comprehensive and coordinated global responses.

The term ‘new normal’ gained popularity as it encapsulated the notion that the pandemic had triggered profound changes, necessitating rapid adaptation from the global community. It emphasized the reality that encountering multiple and concurrent challenges in the face of the pandemic had become typical [9]. The *Lancet's* Commission on Lessons for the future from the COVID‐19 pandemic extensively underscores the significance of recognizing the manifold challenges brought about by the pandemic and the need to embrace and adapt to the radical changes it has triggered [10]. Furthermore, it stresses the importance of making informed choices and decisions to successfully navigate this new normal. In 2020, the emphasis was placed on implementing social mitigation measures to contain the spread of the virus. However, with the advent of vaccines, the focus shifted in 2022 towards adapting to the ‘new normal’ and making informed decisions for success [11]. The discontent expressed by citizens in response to China's stringent pandemic restrictions, driven by the zero‐COVID policy in late 2022, underscored the importance of global communities maintaining adaptability and fostering innovation in the face of challenges.

## CHALLENGES AND OPPORTUNITIES OF THE ‘NEW NORMAL’

The term ‘new normal’ permeated every facet of society, including education, healthcare, economics and politics. In the realm of education, the shift towards digitalization became evident, with hybrid and blended synchronous meetings becoming increasingly prevalent. Unlike the pre‐pandemic era, where physical university campuses were the primary educational spaces, the activation of emergency remote teaching during the pandemic necessitated a rapid focus on digital platforms [12]. Within a remarkably short period, teachers transformed lessons from traditional classroom settings into their digital counterparts. However, the lack of face‐to‐face interactions resulted in heightened psychological concerns among students [12]. As pandemic restrictions were lifted, the ‘new normal’ phase became a prevailing paradigm in the diverse sectors. However, society exhibited divergent approaches with the development of this phase. Although many readily embraced the convenience of remote work and studying from home, preferring to avoid commuting, others resisted and yearned for a return to pre‐pandemic norms [13].

The COVID‐19 pandemic revealed deep structural inequalities in the economy, both locally and globally. The economic consequences of the pandemic have driven further divergence between rich and poor countries. Some stakeholders were hesitant in the push for the return to pre‐pandemic life, fearing the return of pre‐pandemic behaviours and the exacerbation of health disparities [14]. Although vaccination rates were increasing, there was the risk of generalizing the ‘new normal’ too quickly and normalizing the behaviours that were problematic before and during the pandemic. The concept of the ‘new normal’ also increased the risk of overlooking the experiences of marginalized groups and ignoring societal issues, such as homelessness, poverty and systemic health disparities [14].

Nevertheless, the ‘new normal’ provides opportunities for the long‐term institutionalization of beneficial measures, such as digitalization, improved surveillance and public preventive measures. The relationship between people and technology is expected to deepen across all sectors, including healthcare and social interactions [15]. Advancements, such as virtual reality, augmented reality and artificial intelligence, can enable individuals to live smarter, safer and more productive lives. Public health decision‐making can evolve into real‐time data‐driven decision‐making and knowledge translation, empowering stakeholders to make more informed choices. Additionally, the ‘new normal’ has emphasized the importance of mental health, wellness and mindfulness. Work‐life balance has been disrupted since the start of the pandemic, leading to some workplace trends, such as high labour turnover and ‘quiet quitting’, that gained ground [16]. The blended phase emerged in the educational sector at a ‘new normal’ state where academics continue to discover novel and improved approaches to designing lessons and sharing knowledge [12].

## THE FUTURE OF THE ‘NEW NORMAL’

Moving forward, it is crucial to address the challenges of the new normal while simultaneously capitalizing on the opportunities it presents. In gearing up the global community for responsiveness and resilience, health systems should develop the value of learning from experiences. Lessons learned from the ‘new normal’ should identify what has changed and what has not changed across sectors. By doing so, we can create a healthy environment different from the past but no less rich with possibilities for those who are prepared. To ensure success, choices for the ‘new normal’ and beyond should reflect responsiveness and resilience, particularly in healthcare and society. Health systems must develop strategies, goals and benchmarks for public health planning and response systems. This includes the deployment and utilization of real‐time health information infrastructure, flexible health systems with a dedicated implementation workforce, enhancing trust in government and public health institutions and a collective belief in the public good. Moreover, to address health and ensure equitable access to resources and address health disparities, public health policies must be designed with marginalized and vulnerable populations in mind.

Rebuilding trust in public health institutions and collective action in service of public health is crucial for the success of the future ‘new normal’. This can be achieved by instituting necessary reforms that detail a continuum of strategic plans for and with the new normal and improving health systems. Efforts to prioritize mental health, wellness and mindfulness should be integrated into public health policies and programs, including support for individuals at the workplace. Policymakers should specify the goals and strategies for the ‘new normal’ and communicate these clearly to the public. Doing so can rebuild trust, create a more equitable society and be better prepared for future challenges.

## CONCLUSIONS

The ‘new normal’ was a crucial concept in 2022, reflecting the significant impact of the COVID‐19 pandemic on society and global health. It has brought about substantial changes, presenting challenges and opportunities. It is vital to learn from the experiences of the ‘new normal’ to create a responsive and resilient community. The public health community must remain adaptive and innovative, embracing the lessons of the ‘new normal’ in preparing and responding to future challenges.

## AUTHOR CONTRIBUTIONS

*Conceptualization; data curation; formal analysis; writing original draft; writing review editing*: Don Eliseo Lucero‐Prisno III. *Data curation; project administration; writing – original draft; writing – review and editing*: Deborah Oluwaseun Shomuyiwa. *Formal analysis; writing – original draft; writing – review and editing*: Sheena Ramazanu, Yusuff Adebayo Adebisi. *Writing – review and editing*: Jude Altiveros Duarte, Patrick Alain Azanza, Martin C. S. Wong, M.B.N. Kouwenhoven.

## CONFLICT OF INTEREST STATEMENT

Don Eliseo Lucero‐Prisno III, Martin CS Wong and M.B.N. Kouwenhoven are Editorial Board members of Public Health Challenges and are co‐authors of this article. Deborah Oluwaseun Shomuyiwa, Yusuff Adebayo Adebisi and Sheena Ramazanu are Youth Editorial Board members of Public Health Challenges and co‐authors of this article. To minimize bias, they have been excluded from all editorial decision‐making related to the acceptance of this article for publication.

## FUNDING INFORMATION

There is no funding for the development of this paper.
